# Interplay between estrogen receptor and AKT in Estradiol-induced alternative splicing

**DOI:** 10.1186/1755-8794-6-21

**Published:** 2013-06-11

**Authors:** Poornima Bhat-Nakshatri, Eun-Kyung Song, Nikail R Collins, Vladimir N Uversky, A Keith Dunker, Bert W O’Malley, Tim R Geistlinger, Jason S Carroll, Myles Brown, Harikrishna Nakshatri

**Affiliations:** 1 Departments of Surgery, Indiana University School of Medicine, 980 West Walnut Street, Indianapolis, IN 46202, USA; 2 Biochemistry and Molecular Biology, Indiana University School of Medicine, Indianapolis, IN, 46202, USA; 3 Center for Computational Biology and Bioinformatics, Indiana University School of Medicine, Indianapolis, IN 46202, USA; 4 Department of Molecular and Cellular Biology, Indiana University School of Medicine Program in Bioinformatics, School of Informatics, Indiana University, Indianapolis, IN 46202, USA; 5 Department of Medical Oncology, Baylor College of Medicine, Houston, TX 77030, USA; 6 Dana-Farber Cancer Institute, Boston, MA 02115, USA; 7 Current address: Department of Molecular Medicine, University of South Florida, College of Medicine, 12901 Bruce B. Downs Blvd, Tampa, FL 33612, USA; 8 Amyris, Emeryville, CA 94608, USA; 9 Cambridge Research Institute and Department of Oncology, Cancer Research UK, University of Cambridge, Robinson Way, Cambridge, CB2 0RE, UK

## Abstract

**Background:**

Alternative splicing is critical for generating complex proteomes in response to extracellular signals. Nuclear receptors including estrogen receptor alpha (ERα) and their ligands promote alternative splicing. The endogenous targets of ERα:estradiol (E2)-mediated alternative splicing and the influence of extracellular kinases that phosphorylate ERα on E2-induced splicing are unknown.

**Methods:**

MCF-7 and its anti-estrogen derivatives were used for the majority of the assays. CD44 mini gene was used to measure the effect of E2 and AKT on alternative splicing. ExonHit array analysis was performed to identify E2 and AKT-regulated endogenous alternatively spliced apoptosis-related genes. Quantitative reverse transcription polymerase chain reaction was performed to verify alternative splicing. ERα binding to alternatively spliced genes was verified by chromatin immunoprecipitation assay. Bromodeoxyuridine incorporation-ELISA and Annexin V labeling assays were done to measure cell proliferation and apoptosis, respectively.

**Results:**

We identified the targets of E2-induced alternative splicing and deconstructed some of the mechanisms surrounding E2-induced splicing by combining splice array with ERα cistrome and gene expression array. E2-induced alternatively spliced genes fall into at least two subgroups: coupled to E2-regulated transcription and ERα binding to the gene without an effect on rate of transcription. Further, AKT, which phosphorylates both ERα and splicing factors, influenced ERα:E2 dependent splicing in a gene-specific manner. Genes that are alternatively spliced include FAS/CD95, FGFR2, and AXIN-1. E2 increased the expression of FGFR2 C1 isoform but reduced C3 isoform at mRNA level. E2-induced alternative splicing of FAS and FGFR2 in MCF-7 cells correlated with resistance to FAS activation-induced apoptosis and response to keratinocyte growth factor (KGF), respectively. Resistance of MCF-7 breast cancer cells to the anti-estrogen tamoxifen was associated with ERα-dependent overexpression of FGFR2, whereas resistance to fulvestrant was associated with ERα-dependent isoform switching, which correlated with altered response to KGF.

**Conclusion:**

E2 may partly alter cellular proteome through alternative splicing uncoupled to its effects on transcription initiation and aberration in E2-induced alternative splicing events may influence response to anti-estrogens.

## Background

Estradiol (E2) signaling primarily involves activation of nuclear receptors, estrogen receptors alpha (ERα) and beta (ERβ), which function as transcription factors that regulate gene expression through either DNA binding or through protein-protein interaction [1,2]. E2 signaling in cells is further controlled by several post-transcriptional modifications of ERα and ERβ including phosphorylation, acetylation, and ubiquitination. These post-transcriptional events influence the ability of ERα to interact with co-regulator molecules, its stability, and localization. Kinases known to phosphorylate ERα include MAPK, IKKα, RSK, AKT/PKB, p38 kinase, PKA, Src, cyclin A/cdk2, and cdk7 [1,3-7]. It is suggested that changes in the phosphorylation status of the receptor contribute to ERα dysfunction in various pathological conditions including breast cancer.

Alternative splicing is an important post-transcriptional mechanism that permits the generation of multiple protein products from a single gene. 92-94% of human genes undergo alternative splicing and 70-90% of these spliced RNAs are translated into proteins [8,9]. A recent genome-wide sequencing indicated 22,000 tissue-specific alternative splicing events [8]. Two families of splicing factors have been identified: heterogeneous nuclear ribonucleoprotein (hnRNP) related proteins and the serine-arginine rich (SR) proteins [9,10]. Phosphorylation controls activity of these proteins [9,10]. For example, RS domains of SR proteins contain multiple copies of consensus AKT phosphorylation site RXRXXS and AKT controls their activity in the nucleus and cytoplasm [11].

Defects in alternative splicing are linked to various diseases including spinal muscular atrophy, neurofibromatosis type 1, cystic fibrosis, breast cancer, and ovarian cancer [12,13]. For example, alternative splicing can change CC3, which codes for a protein with anti-metastatic and pro-apoptotic properties, to TC3, which codes for a protein with pro-metastatic and anti-apoptotic properties [14]. Alternative splicing of the transcription factor FOXP1 influences pluripotency and differentiation of embryonic stem cells [15]. At least 15% of human genetic diseases arise from mutations either in consensus splice sites or in splicing silencer or enhancer elements [12]. Cancer-associated alternative splicing, which is regulated by FOX1, FOX2, and Nova proteins, has been reported [16,17]. These alternative splicing events empower cancer cells to express developmentally regulated proteins [13]. Breast cancer subtypes show distinct splicing pattern, which may partly be related to FOX1/FOX2 expression [16,18]. Genetic alterations in splicing machinery is linked to myelodysplasia [19]. Recent studies using bioinformatics tools including intrinsic disorder predictions have shown that alternative splicing is a non-random event and often involves the region of the protein engaged in protein-protein interaction [20]. Thus, loss or gain of exons through alternative splicing has major functional implications.

A clear link between steroid regulated transcription and alternative splicing has been established [21]. At least 25 proteins have been described to have both transcription coregulator and splicing activity [22]. Proteins involved in steroid-regulated alternative splicing events include U2AF65-related proteins CAPERα and CAPERβ, ASC-1, ASC-2, and CoAA [23]. Several of these molecules interact directly with ERα. ERα:E2 controls transcription-coupled alternative splicing of the first intron in a gene specific manner, which impacts the rate of co-transcriptional RNA processing [24]. However, whether these ERα:splicing factor interactions contribute to breast cancer subtype-enriched alternative splicing or whether ERα:E2 induces alternative splicing uncoupled to transcription initiation activity, is yet to be determined. Additionally, there are limited number of reports that have examined the effect of ERα phosphorylation on alternative splicing [25]. ERα phosphorylated at S118 by MAPKs interacts with the splicing factor SF3a, a component of U2 snRNP complex, and promotes exon skipping [25].

We had previously shown the effects of AKT on ERα:E2 mediated transcription, which correlated with the phosphorylation of S167 residue of ERα [6]. Crosstalk between ERα and AKT is likely to play a significant role in ERα-positive breast cancer because recent genome analyses have observed frequent activating PI3K mutation in ERα-positive breast cancer, which often leads to increased AKT activity [26,27]. Using chromatin immunoprecipitation coupled microarray (ChIP-on-chip) and gene expression analysis, we demonstrated specific effects of AKT on ERα binding to the genome and E2-regulated transcription [28]. The effect of AKT on E2-induced alternative splicing is unknown. To test the effect of AKT on E2-induced alternative splicing, we first performed CD44 minigene splicing assay and observed a dominant effect of AKT on E2-induced alternative splicing in two cell types examined. To identify endogenous targets of E2-regulated alternative splicing and the influence of AKT on this process, we performed ExonHit Apoptosis Splice Array analysis of ERα-positive/E2-dependent MCF-7 breast cancer cells (MCF-7p) and the same cell line overexpressing constitutively active AKT (MCF-7AKT) with or without E2 treatment. Combined analysis of splice array and other published cancer-specific splicing data with E2-induced ERα cistrome and gene expression arrays revealed two distinct categories of E2-regulated alternatively spliced genes [17,28-30]. E2 induced alternative splicing of FAS/CD95 in MCF-7 cells, which correlated with resistance to FAS-activation induced apoptosis. In addition, E2 increased the expression of fibroblast growth factor receptor 2 (FGFR2) C1 isoform without significantly altering C2 isoform levels in MCF-7p cells. Consistent with the effect of E2 on FGFR2 splicing, MCF-7 cells showed differential response to keratinocyte growth factor (KGF), a ligand for FGFR2 C1 and C2 isoforms, in the presence of E2 and tamoxifen, an anti-estrogen. Furthermore, acquired resistance to anti-estrogens tamoxifen and fulvestrant was associated with distinct expression pattern of FGFR2 isoforms and response to KGF. Collectively, these results suggest a role for E2 induced alternative splicing in anti-estrogen response of breast cancer cells.

## Results

### AKT influences E2 induced alternative splicing

Apart from SR proteins that are phosphorylated by AKT [11], the RS domain of splicing factors CAPERα and CAPERβ contain several putative AKT phosphorylation sites [23]. Both CAPERα and CAPERβ associate with ERα and modulate E2-dependent transcription as well as alternative splicing [23]. Thus, AKT can potentially influence E2-induced alternative splicing by phosphorylating multiple components of the splicing machinery. We used the CD44 minigene splicing system containing variable exons 4 and 5 (v4,5) of CD44 gene under the control of estrogen response element driven promoter (ERE-CD44) and measured the exon inclusion/skipping to determine the effect of AKT on E2-induced alternative splicing [21]. Two cell systems were used: 1) 293 cells requiring transfection of both the CD44 minigene and ERα or its mutants; 2) MCF-7p and MCF-7AKT cells transfected with the CD44 minigene. Studies in 293 cells allowed us to study phosphorylation defective mutants of ERα on splicing as these cells lack ERα. Studies in MCF-7 cells allowed us to study E2-induced splicing in the context of E2 responsive breast cancer cells, endogenous ERα and to distinguish ERE-dependent and ERE-independent effects of E2 on alternative splicing. We designed unique primers spanning exon-exon junctions to measure exon-included and exon-skipped products by quantitative reverse transcription polymerase chain reaction (qRT-PCR).

**Figure 1:**
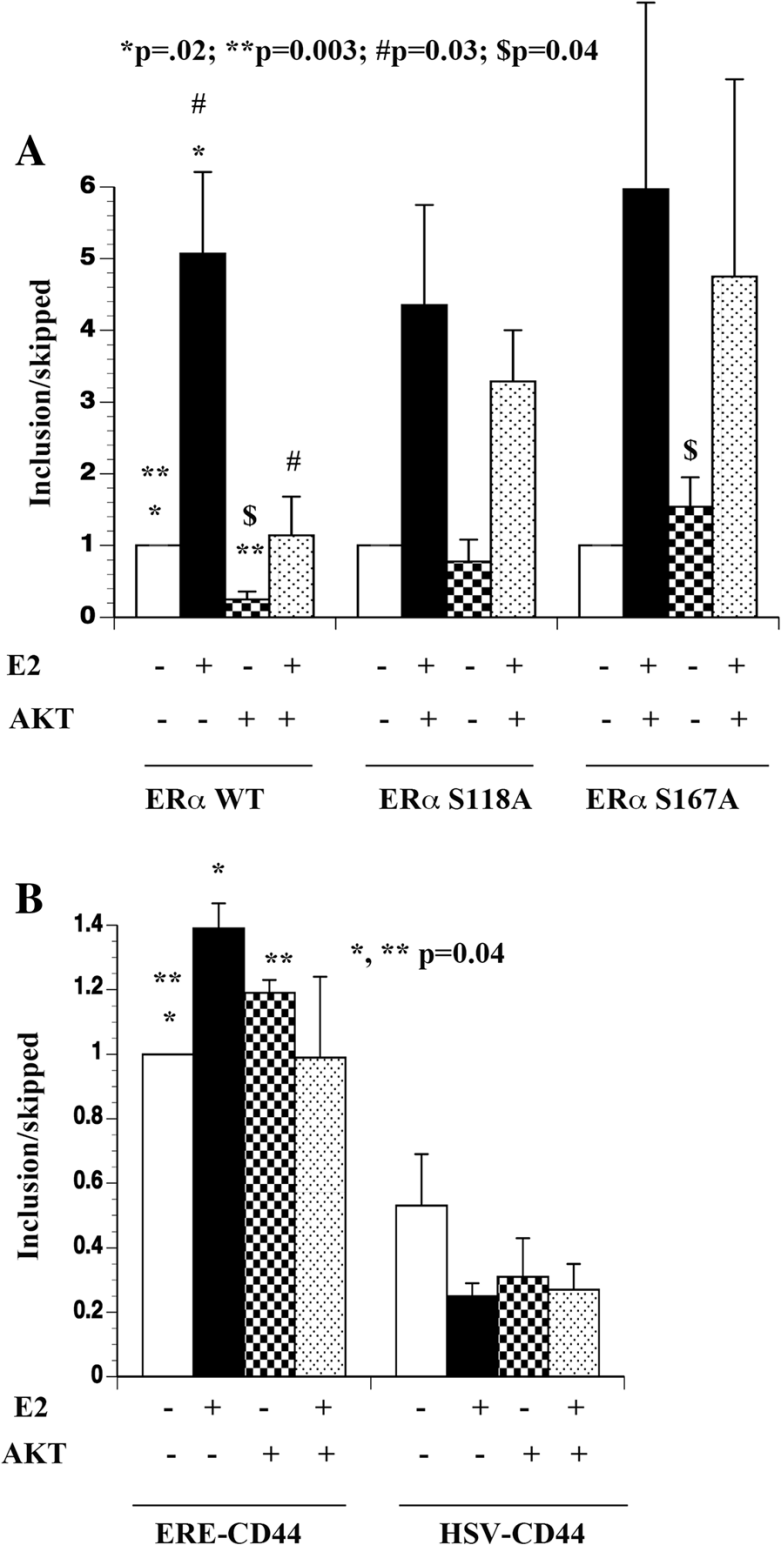
**The effect of AKT on E2-induced alternative splicing of CD44 minigene. A**) CD44 minigene splicing assay in 293 cells. 293 cells were transfected with ERE-CD44 minigene (2 μg), indicated ERα constructs (2 μg) and either vector control pCDNA3 or pCDNA3-CA-AKT expression vector (0.5 μg). A day after transfection, cells were treated with ethanol or E2 for 24 hours. qRT-PCR was performed using primers that amplify exon-included or exon-skipped products. The ratio between exon-included and exon-skipped products in untreated ERα and CD44-ERE transfected cells was normalized to one and relative change in the ratio is presented. Ratio of >1 indicates elevated exon-inclusion whereas a ratio of <1 indicates enhanced exon-skipping. **B**) The effect of E2 on CD44 minigene splicing in MCF-7p and MCF-7AKT cells. Cells were transfected with ERE-CD44 minigene or HSV-CD44 minigene and assays were performed as in A. Statistically significant differences are indicated.

In 293 cells, only exon skipped transcripts from ERE-CD44 minigene could be reliably measured in the absence and presence of E2 by qRT-PCR (exon-included CT >30, exon-skipped ~25). In contrast, robust ERE-CD44 gene activity with expression of both exon-included (~27 CT) and exon-skipped products (~24 CT) was observed when co-transfected with ERα (Figure 1A). The exon-inclusion/exon-skipped ratio changed dramatically in cells treated with E2 for 24 hours (range 1.7 to 6 fold in favor of included product, Figure 1A). We next compared E2-induced alternative splicing in the presence of MAPK-phosphorylation defective (S118A) and AKT phosphorylation defective (S167A) mutants of ERα. ERα mutants were effective as wild type ERα in modulating alternative splicing in the presence of E2 (Figure 1A).

We then compared ERα:E2-induced alternative splicing in the presence of constitutively active AKT. AKT had a dominant effect on alternative splicing of ERE-CD44 minigene-derived transcripts. Compared to control vector-transfected cells, exon-included/exon-skipped transcript ratio was dramatically lower indicating the ability of AKT to promote exon skipping and E2 failed to reverse this effect of AKT (Figure 1A, p=0.003). Please note that AKT did not influence transcription of ERE-CD44 minigene in the absence of ERα, as only exon-skipped transcripts under this transfection condition could be reliably measured (data not shown). Interestingly, AKT failed to influence alternative splicing events orchestrated by ERα phosphorylation defective mutants. In particular, the ability of AKT to promote exon skipping was completely lost in cells transfected with S167 mutant, which is defective in AKT-mediated phosphorylation (p=0.04; exon inc/skip ratio in wild type vs S167A mutant ERα transfected cells with AKT). These results indicate that AKT alters ERα:E2 induced alternative splicing in ERα S167 phosphorylation-dependent manner to a larger extent and S118 to a minor extent.

We performed similar assay in parental MCF-7 cells (MCF-7p) and cells overexpressing AKT (MCF-7AKT) with ERE-CD44 minigene. Additionally, we included CD44 minigene driven by herpes simplex virus enhancer-promoter (HSV-CD44) to account for secondary effects of ERα:E2 on alternative splicing. As with 293 cells, E2 increased exon-inclusion/exon-skipped ratio in cells transfected with ERE-CD44 minigene (Figure 1B). Although alternative splicing of HSV-CD44 minigene in E2 treated cells was different from untreated cells, none of the differences reached statistical significance (Figure 1B). Similarly, AKT had minimum effect on alternative splicing of transcripts from HSV-CD44 minigene (Figure 1B). Taken together, these results suggest that E2 has distinct effects on alternative splicing of genes with ERE compared to genes without ERE. In addition, AKT has dominant effect on E2-induced alternative splicing or confers ligand-independent splicing activity to ERα, depending on the cell type.

### E2 induced alternative splicing of apoptosis-related genes

To identify endogenous targets of E2-induced alternative splicing and the potential influence of AKT in this process, we performed microarray analysis that detects alternative splicing of 893 genes related to apoptosis. Array analysis was performed in quadruplicate using RNA from MCF-7p and MCF-7AKT cells with or without E2 treatment for three hours. Three hours time point was selected to enrich for genes that are primary targets of E2-regulated transcription and alternative splicing. Data from both cell types together with or without E2 treatment were analyzed using a more comprehensive ANOVA model after dye bias and cell type effects were removed. Four hundred sixty three splicing events out of possible 10659 splice events (p<0.001) (~4%) targeting 154 genes (17% of genes) were altered in E2 treated cells compared to untreated cells. These events were common to both cell types. Individual cell types also showed E2-dependent changes in splicing patterns (527 in MCF-7p and 451 in MCF-7AKT) although statistical analysis is not as robust as combined analysis because comparison was only between two cell types (with and without E2 treatment). A gene list is provided as Additional file 1, Additional file 2, Additional file 3 available online and a partial list of the genes, few of which were verified further by qRT-PCR assays, is provided in Table 1 along with type of splicing events and fold change in response to E2 treatment.

**Table 1:** Selected E2-induced splicing events

**Gene name**	**Reference**	**Variant**	**Splicing event**	**Long form**	**p value**	**Fold change with E2**
Caspase 7, apoptosis-related cysteine protease (CASP7), transcript variant beta,	NM_033340	BM459840	ASA	R	0.000	2.01
		BG339524	ES	R	0.000	2.20
		BX458514	ES	R	0.000	1.96
		AU131461	ASD	R	0.000	2.21
		BX443602	ASD	V	0.000	2.18
Axin 1 (AXIN1), transcript variant 1	NM_003502	AA954457	ES	R	0.000	1.48
		BU500157	ES	R	0.000	1.48
Cyclin E1 (CCNE1), transcript variant 1	NM_001238	BG395682	ES	R	0.000	1.41
		CR601172	IR	V	0.000	1.57
		BE887210	ES	R	0.001	1.41
Homeodomain interacting protein kinase 2 (HIPK2)	NM_022740	AF326592	ASA	R	0.000	1.36
Tumor necrosis factor receptor superfamily, member 12A (TNFRSF12A, FN14)	NM_016639	BC064377	ES	R	0.000	-1.4
Tumor necrosis factor receptor superfamily, member 10A (TNFRSF10A)	NM_003844	BI029612	ASD	R	0.001	-2.01
Inhibitor of DNA binding 1, dominant negative helix-loop-helix protein (ID1), transcript variant 2	NM_181353	AI767705	PIED	R	0.000	-2.52
		BP261920	PIED	R	0.000	-2.71
Tumor necrosis factor receptor superfamily, member 6 (TNFRSF6), transcript variant 1 (FAS)	NM_000043	NM_152876	ES	R	0.000	1.14
Tumor necrosis factor receptor superfamily, member 6 (TNFRSF6), transcript variant 1 (FAS)	NM_000043	NM_152874	ES	R	0.001	-1.36
Ubiquitin-activating enzyme E1C (UBA3 homolog, yeast) (UBE1C), transcript variant 1	NM_003968	AV716795	ASA	R	0.000	3.85

Reference and variant transcripts with accession number in the array are shown. Longer version of the transcript in the array (reference or the variant) is also indicated. Positive number in fold change column indicates E2-induced splicing generating higher levels of the variant whereas negative number indicates repression of splicing event leading to generation of the variant. ASA, alternative splice acceptor; ASD; Alternative splice donor; ES, exon skipping; IR, intron retention; PEID, partial internal exon deletion. Additional splicing events including novel exons (NE) are in Additional file 1, Additional file 2, Additional file 3.

### Genes that undergo E2-induced alternative splicing are enriched for ERα binding sites

E2 induced alternative splicing could involve E2-regulated transcription-coupled splicing, ERα binding to the gene that permits co-recruitment of splicing factors without an effect on the rate of transcription initiation, or a secondary event due to E2-mediated induction/repression of a splicing factor(s). To test these possibilities, we examined alternatively spliced genes for ERα binding sites from our previous ChIP-on-chip study datasets of MCF-7p and MCF-7AKT cells as well as a published study involving ChIP-seq of untreated, E2-treated or tamoxifen treated MCF-7 cells [28,29]. Among 154 genes that underwent alternative splicing in E2 treated cells, 89 genes contained ERα binding sites in regions within or ~20-kb 5’ or 3’ of the coding unit (Additional file 1). A 20-kb window has previously been shown to be optimal for ERα enhancer-promoter interactions [31]. However, we cannot rule out the possibility of ERα binding sites located at 50–100 KB distance from 5’ and 3’ of the coding units and ERα binding to these sites influencing gene expression or alternative splicing through looping mechanism. We had previously shown that 299 of 837 E2-regulated genes in MCF-7p cells and 363 of 1063 E2-regulated genes in MCF-7AKT cells (~35%) contain ERα binding sites [28]. In comparison, it appears that ERα binding sites are enriched on genes that underwent E2-induced alternative splicing (89 out of 154, ~60%, p=0.0001, Fisher’s exact test).

**Figure 2:**
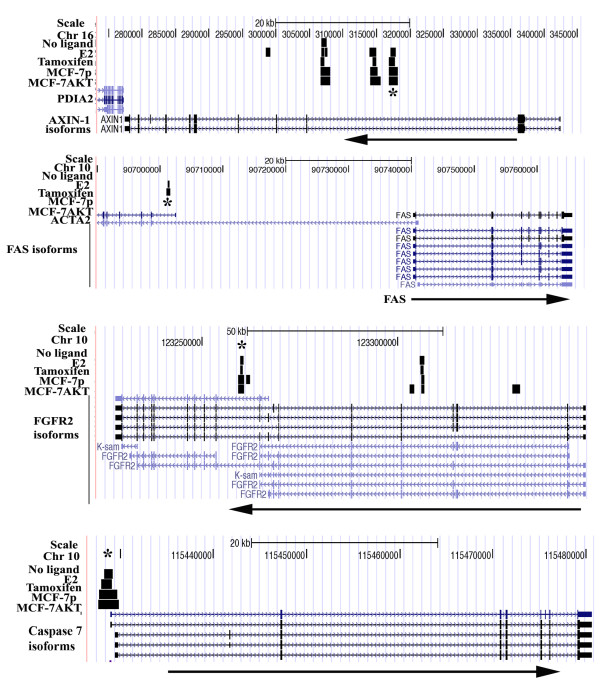
**ERα binding pattern to alternatively spliced genes in response to E2 treatment.** ChIP-on-chip data of MCF-7p and MCF-7AKT cells and/or ChIP-seq of MCF-7 cells with or without E2 or tamoxifen treatment reveal ERα binding sites within or around alternatively spliced genes. Relative position of genes on respective chromosomes along with ERα binding site detected in treated cells (black bar) are shown. Black arrow indicates direction of the gene. Asterisk indicates ERα binding sites verified by ChIP-qPCR.

**Figure 3:**
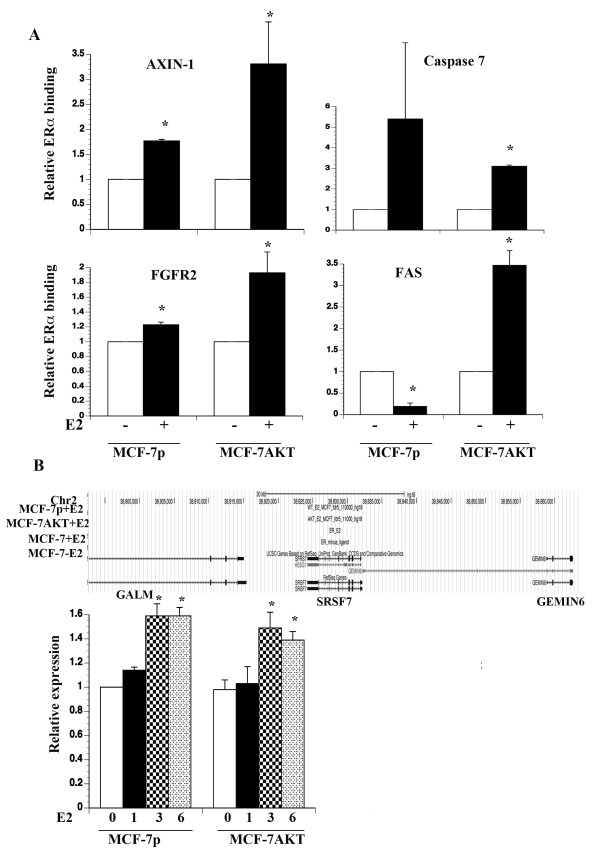
**Verification of ER**α **binding to alternatively spliced genes. A**) ChIP-qPCR was used to confirm ERα binding to genomic regions of AXIN-1, Caspase 7, FGFR2, and FAS genes. Relative enrichment of ERα binding upon E2 treatment is shown (average and standard error of the mean). Data from one hour (AXIN-1 and FGFR2), two hours (Caspase 7), and four hours (FAS) after E2 treatment are shown. Asterisks indicate significant enrichment or loss of binding (p<0.02) upon E2 treatment. **B**) E2 increases SRSF7 expression in both MCF-7p and MCF-7AKT cells. Mean ±SE is shown. *p<0.05, untreated versus E2 treated. The chromosomal region encompassing SRSF7 gene lacks ERα binding sites based on our ChiP-on-chip as well as ChIP-seq data published by others (top).

ERα binding pattern to AXIN-1, Caspase 7, FGFR2, and FAS, which were alternatively spliced upon E2 treatment, is shown in Figure 2. FGFR2 displayed unique ERα binding sites in cells overexpressing AKT and one of these sites is located in intron 2. This intron contains binding sites for several transcription factors and single nucleotide polymorphism in this intron is associated with breast cancer susceptibility, particularly postmenopausal breast cancers, which are usually ERα-positive [32-34]. Although our ChIP-on-chip did not identify ERα binding sites around FAS, ChIP-seq data from the other study [29] showed an ERα binding site ~40 kb upstream of the transcription start site. We performed ChIP assay with ERα antibody followed by qPCR to confirm ERα binding to select binding sites of the above genes (Figure 3A). Although assays were performed at three time points after E2 treatment (one, two, and four hours after E2 treatment), time points that showed significant ERα binding differences between untreated and E2 treated conditions are shown in the figure. With the exception of FAS, statistically significant increase in ERα binding to specific regions of the above genes upon E2 treatment was observed in MCF-7p, MCF-7AKT, or both cell types. E2 had distinct effect on ERα binding to FAS gene in MCF-7p and MCF-7AKT cells. Unliganded ERα bound to previously identified ChIP-seq region in FAS gene in MCF-7p cells, which was reduced upon E2 treatment. However, in MCF-7AKT cells, E2 increased ERα binding, which correlated with repression of E2-regulated expression of total FAS transcripts in these cells (see below). ChIP with control IgG did not display any E2-dependent increase in amplification of ERα binding regions (data not shown). Taken together, the results presented above demonstrate a correlation between ERα binding to genes whose transcripts undergo alternative splicing in response to E2 treatment.

We used two resources to determine whether E2-induced alternative splicing is coupled to ERα binding as well as changes in overall rate of transcription. First, we determined the effect of E2 on overall mRNA levels of alternatively spliced genes with ERα binding sites in MCF-7p or MCF-7AKT cells using gene expression array data from same cells treated with E2 for four hours [28]. Among 89 alternatively spliced genes with ERα binding sites, E2 induced the expression of eight genes but repressed the expression of two genes in MCF-7p cells. In MCF-7AKT cells, E2 increased the expression of 12 genes but repressed the expression of four genes (Additional file 1). Therefore, ~80% of genes with ERα binding sites undergo alternative splicing upon E2 treatment without changing overall levels of transcription. The second set of data derived from the publicly available database (http://www.nursa.org/gems/) was used to independently verify this possibility. This database contains gene expression meta-signature of MCF-7 cells treated with E2 for three hours and 24 hours, which permitted us to measure early and late effects of E2 on overall transcription of alternatively spliced genes [30]. Among 89 genes with ERα binding sites, 18 genes were early-response inducible genes (3–4 hours) whereas 15 genes were late-response inducible genes (24 hours) with q-value of <0.05 (Additional file 1, Additional file 4). 16 and 22 genes were repressed at early and late time points, respectively. Remaining genes with ERα binding sites (60%) underwent alternative splicing without an effect on overall transcription after E2 treatment. Among 65 alternatively spliced genes without ERα binding sites, 13 and 19 genes were early and late E2 induced genes, respectively, whereas four and 10 were early and late repressed genes, respectively. Based on these two independent analyses, we conclude that E2 induced alternative splicing need not have to be coupled to the effects of E2 on the rate of transcription initiation. In fact, E2 had no effect on transcription of the majority of genes (85 out of 154 were unaffected at the level of transcripts) that underwent E2-regulated alternative splicing with or without ERα binding sites.

### E2 regulated expression of splicing factors

Since ~40% of genes lacking ERα binding sites within 20 kb of 5’ and 3’ ends of the coding unit underwent E2 dependent alternative splicing, we examined the effect of E2 on the expression of genes of the alternative splicing machinery including those linked to cancer. First, we analyzed ChIP-on-chip for ERα binding and gene expression array data of E2 treated MCF-7p and MCF-7AKT cells for E2-regulated expression of splicing factors [28] and observed E2-dependent increase in the SRSF7 (also called 9G8) by more than 2-fold. Indeed, E2 increased the expression of SRSF7 in both MCF-7p and MCF-7AKT cells by more than 2-fold, despite lacking ERα binding sites based on ChIP-on-Chip and ChIP-seq data (Figure 3B). Therefore, SRSF7 may contribute to E2-regulated alternative splicing of genes without ERα binding sites. FOX2 (also called RBM9) downregulation is responsible for cancer-associated alternative splicing [16]. However, FOX2 gene lacks ERα binding sites and E2 had no effect on its expression in MCF-7p and MCF-7AKT cells [28]. Genome-wide RNAi screen for genes involved in alternative splicing of apoptosis-related genes revealed ASF/SF2/SRSF1 as the main splicing factor that regulates apoptosis [35]. The regulatory regions of SF2 contain an ERα binding site; however, E2 did not alter the expression of this gene in both MCF-7p and MCF-7AKT cells. The regulatory regions of Polypyrimidine Tract Binding protein 1 (PTB), another cancer-associated splicing factor [13], bind to ERα in tamoxifen but not E2 treated cells and E2 induces its expression only after 24 hours. Thus, PTB is less likely to be a major player in E2-induced alternative splicing. The regulatory regions of SRp20, another splicing factor overexpressed in cancer [36], contain ERα binding sites and SRp20 is induced by E2 at transcript level (1.36-fold, q value <0.01). However, we did not observe elevated SRp20 protein levels in cells treated with E2 compared to untreated cells possibly due to higher abundance and stability of the protein (data not shown). Furthermore, analysis of more than 20 known splicing factors did not reveal significant effects of E2 on their expression [37,38]. Thus, SRSF7 is the only documented E2-inducible splicing factor, which may cause alternative splicing as a secondary event in E2 treated cells.

### Characterization of E2-induced alternative splicing at mRNA level

**Figure 4:**
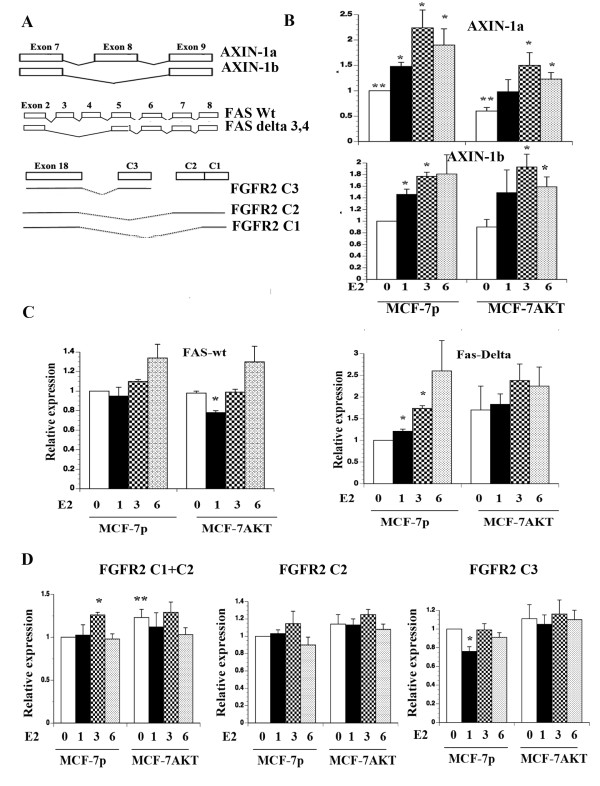
**E2-induced alternative splicing of endogenous genes. A**) Partial exon-intron structure of genes that were alternatively spliced upon E2 treatment. Exon-intron structure of wild type and spliced variants are shown. **B**) qRT-PCR analyses of AXIN-1a and AXIN-1b in untreated and E2 treated MCF-7p and MCF-7AKT cells. Statistically significant increase in AXIN-1a and AXIN-1b in E2 treated cells compared to untreated cells is indicated (p<0.05). Down-regulation of AXIN-1a expression in MCF-7AKT cells compared to MCF-7p cells is indicated (**p=0.04). **C**) E2 increases the levels of Δ3,4 FAS but not total FAS. qRT-PCR was performed using primers that amplify the common exon 9 (for total FAS) or spanning exons 2 and 5 junction and exon 6. *p<0.02 untreated versus E2 treated condition. **D**) The effect of E2 and AKT on alternative splicing of FGFR2. E2 increased the expression of C1 isoform (p=0.0001) but reduced C3 levels in MCF-7p cells (p=0.004). Basal FGFR2 C1 levels were elevated in MCF-7AKT cells compared to MCF-7p cells (p=0.05).

A schematic view of some of the genes that underwent alternative splicing in response to E2 treatment is given in Figure 4A. Alternative splicing pattern of the FGFR2 C-terminus is also shown. FGFR2 was included in this analysis because it is one among 19 genes that undergo breast cancer-specific alternative splicing, which results in proteins with distinct functional domains [17]. To visualize and measure splice variants, we employed qRT-PCR using primer sets that measure wild type or isoform mRNA levels. We selected AXIN-1, FAS, and FGFR2 for further analysis because of their known role in breast cancer and apoptosis.

AXIN-1a and AXIN-1b showed distinct expression pattern in MCF-7p and MCF-7AKT cells. In MCF-7p cells, E2 increased the expression of both AXIN-1a and AXIN-1b although there was a difference in the expression levels of these isoforms at given time after E2 treatment (Figure 4B). Basal AXIN-1a but not AXIN-1b levels were lower in MCF-7AKT cells compared to MCF-7p cells (p=0.006) suggesting the ability of AKT to alter AXIN-1 splicing independent of ERα and E2. Consequently, overall levels of AXIN-1a but not AXIN-1b were lower in E2 treated MCF-7AKT cells compared to MCF-7p cells. AXIN-1b lacks exon-8, which codes for a 30 amino acid region containing a putative casein kinase I phosphorylation site, oligomerization site, and two potential nuclear export signals [39].

**Figure 5:**
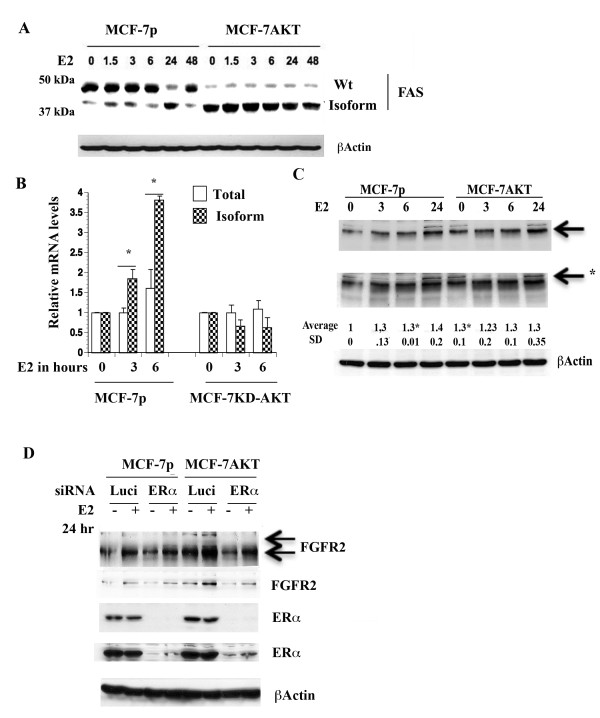
**E2-induced changes in FAS and FGFR2 proteins. A**) MCF-7p and MCF-7AKT cells express FAS proteins of different mobility with or without E2 treatment. Cells were treated with E2 for indicated time (in hours) and cell lysates were subjected to western blotting. **B**) Dominant-negative AKT (KD-AKT) inhibits E2-induced alternative splicing of FAS as evident from qRT-PCR assays. *p<0.03, total vs isoform at 3 and 6 hour E2 treated condition in control cells. **C**) The effect of E2 on FGFR2 protein in MCF-7p and MCF-7AKT cells. *p=0.0004 untreated vs six hour E2 treated MCF-7p cells (for faster migrating FGFR2). *p=0.03 untreated MCF-7p versus untreated MCF-7AKT cells. Two exposures of FGFR2 blot are shown. **D**) ERα is essential for E2 regulated FGFR2 expression. Cells were treated with control luciferase siRNA (Luci) or ERα siRNA. After three days of siRNA transfection, cells were additionally treated with ethanol or E2 for 24 hours. Two exposures of FGFR2 and ERα blots are shown.

Several isoforms of FAS have been characterized [40]. FAS lacking exon 6, which is a more frequent event in cancer, yields a soluble FAS protein with anti-apoptotic activity [13,40]. ExonHit array experiments identified E2-inducible expression of an isoform lacking exons 3, 4, and 6 (NM_152876) but repression of another isoform lacking both exons 4 and 7 (NM_152874) in MCF-7p cells (Table 1 and Additional file 2). We designed multiple qRT-PCR primers to measure the levels of total FAS transcripts (primers spanning exon 9) and splice variants alone. Only primers spanning total FAS and the splice variant lacking exons 3 and 4 (forward primer designed to exon 2 and 5 junction and reverse primer spanning exon 6) gave reproducible results. E2 did not change the levels of total FAS transcripts but significantly increased the levels of the splice variant lacking exons 3 and 4 in MCF-7p cells (Figure 4C) (please see Figure 5 below for even higher induction of the splice variant). E2 decreased the levels of full length but not the splice variant in MCF-7AKT cells after one-hour treatment suggesting a dominant effect of AKT in redirecting ERα to suppress total FAS.

Alternative splicing of FGFR2 generates epithelial cell specific FGFR2 IIIb and mesenchymal cell specific FGFR2 IIIc isoforms [32]. Overall, alternative splicing generates at least 11 splice variants of FGFR2. The C-terminus of FGFR2 IIIb undergoes further alternative splicing to generate constitutively active (C3) and ligand-dependent (C1 and C2) receptors [41-43]. We generated primers that can specifically measure the levels of C1+C2, C2, or C3 transcripts by qRT-PCR and determined the effect of E2 on expression levels of each isoforms in MCF-7p and MCF-7AKT cells. Based on the CT values, C1+C2 transcript is the predominant FGFR2 (~20 CT value) compared to C2 (~26 CT value) or C3 (~29 CT value) in both cell types. RT-PCR assay also confirmed relative abundance of C1 and C2 transcripts compared to C3 transcripts (data not shown). In MCF-7p cells, E2 increased C1+C2 but not C2 transcript to a significant level (p=0.0001, Figure 4D). In contrast, C3 levels were significantly reduced in MCF-7p cells treated with E2 for one hour (p=0.004). C1 levels remain significantly higher compared to C3 levels after three hours of E2 treatment (p=0.0089). The basal C1+C2 was higher in MCF-7AKT cells compared to MCF-7p cells (p=0.05), while the levels of C2 were similar in MCF-7p and MCF-7AKT cells (Figure 4D). In addition, E2 failed to repress C3 levels in MCF-7AKT cells. These effects of AKT on E2-induced splicing of endogenous genes are consistent with the results obtained in transient assays with ERE-CD44 minigene where AKT overexpression recapitulated some of the splicing events that occur in E2 treated cells (Figure 1B). Since AKT decreased the level of specific splice variant in some cases (AXIN-1a for example) but increased splice variants in other instances (FGFR C1+C2, for example), the effects of AKT on alternative splicing of E2-regulated genes is gene-specific but not global. We emphasize that the extent of E2- or AKT-mediated changes in alternative splicing of endogenous genes shown above (30 to 70%) is similar to changes in alternative splicing observed during important biological processes such as embryonic stem cell differentiation (<36% change in FOXP1 alternative splicing) [15].

### The effect of E2 on FAS and FGFR2 proteins

To further confirm E2-induced alternative splicing of above genes leads to measurable expression of distinct protein species, we performed western blotting. These studies were not intended to show a direct correlation between alternatively spliced mRNA and translation of the specific spliced mRNA into a protein. Instead, the purpose was to show that the alternative splicing creates translatable mRNAs. Indeed, E2 treated cells showed a faster migrating FAS protein of ~38 kDa in MCF-7p cells and the basal level of this faster migrating protein was constantly higher in MCF-7AKT cells (Figure 5A). We do note that there were experimental variations in the number of faster migrating bands of FAS in E2 treated cells as well as detection by Western blot analysis possibly reflecting experimental and/or cell lysis conditions altering the stability of the isoforms (data not shown). In addition, since FAS undergoes multiple alternative splicing [40], it is impossible to assign a splice variant transcript to a protein species.

We further characterized the role of AKT in E2-induced alternative splicing of FAS by generating MCF-7 cells overexpressing dominant negative AKT (KD-AKT) [6]. While E2 increased alternatively spliced FAS in parental cells, this effect of E2 was completely abolished in cells overexpressing KD-AKT (Figure 5B).

We observed an effect of E2 on two FGFR2 protein species in MCF-7p cells (Figure 5C). One is an abundantly expressed faster migrating protein, which was induced by E2 (indicated by an arrow). The basal level of this FGFR2 protein was significantly higher in MCF-7AKT cells compared to MCF-7p cells. Another minor slower migrating FGFR2 protein was detected in both cells and levels of this protein declined initially with E2 treatment but restored/increased after 24 hours (indicated by an arrow with star). To determine the requirement of ERα for the expression of these isoforms of FGFR2, we knocked down ERα using siRNA. ERα siRNA significantly reduced basal and E2-inducible expression of both FGFR2 protein species in MCF-7AKT cells (Figure 5D). We are unsure why ERα siRNA had lower effect on FGFR2 expression in MCF-7p cells. Residual ERα in siRNA treated cells may be sufficient for FGFR2 expression in these cells.

### Anti-estrogen resistance is associated with differential FGFR2 C isoform expression

**Figure 6:**
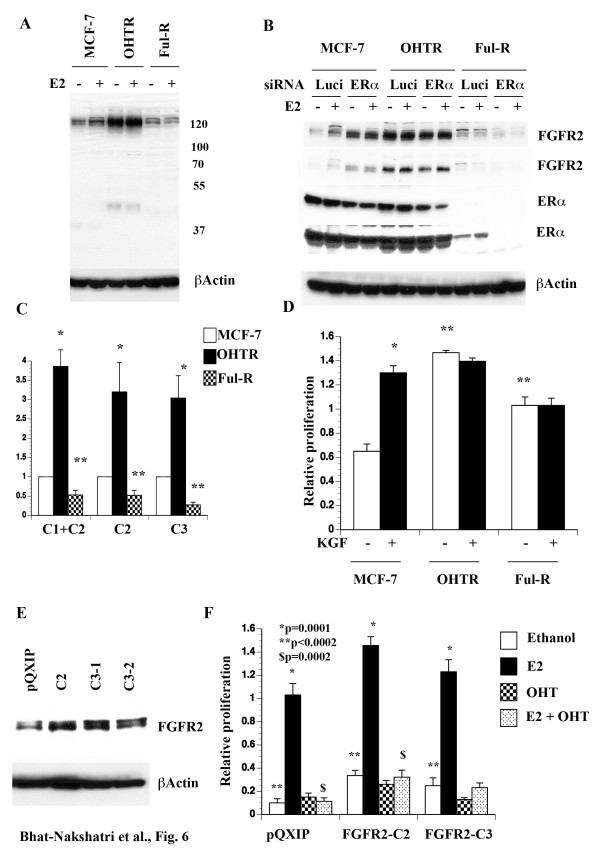
**FGFR2 isoforms and anti-estrogen resistance. A**) FGFR2 proteins in untreated and E2 treated (24 hours) MCF-7, OHTR, and Ful-R cells. Three cell types express different levels of FGFR2 proteins. **B**) FGFR2 C1, C2, and C3 mRNA levels in MCF-7, OHTR, and Ful-R cells. *, ** p<0.05 compared to parental MCF-7 cells. **C**) ERα is essential for the expression of slower migrating FGFR2 protein. siRNA experiments were performed as in Figure 5. Two exposures of ERα blot are shown to demonstrate the presence of residual ERα in Flu-R cells. **D**) The effect of KGF on proliferation of MCF-7, OHTR, and Ful-R cells. Cells were treated with KGF for six days and cell proliferation was measured using BrDU-ELISA. KGF stimulated proliferation of MCF-7 cells but not OHTR or Ful-R cells (*p=0.0001). OHTR and Ful-R cells displayed elevated basal proliferation compared to MCF-7 cells (**p=0.0001). **E**) Western blot analysis shows FGFR2 expression in vector control or FGFR2 C2 or C3 infected cells. **F**) E2 and tamoxifen sensitivity of FGFR2 C2 and C3 overexpressing cells. Cells were treated with E2 (10^-10^ M), 4-hydoxy tamoxifen (1 μM) or both for six days and cell proliferation was measured using BrDU-ELISA. Basal proliferation of FGFR2 C2 and C3 overexpressing cells was significantly higher compared to parental cells. E2 stimulated proliferation of all three-cell types.

We next investigated whether resistance to anti-estrogens is associated with altered expression of FGFR2 isoforms. For this purpose, we used a single cell-derived clone of MCF-7 cell line that has acquired resistance to tamoxifen (OHTR) or fulvestrant (Ful-R). MCF-7 and OHTR cells express similar levels of ERα, whereas Ful-R cells express very little ERα protein compared to other cell types [44]. Western blot analysis was performed to determine whether FGFR2 isoforms are differentially expressed in these cell lines. Similar to results presented in Figure 5, two FGFR2 proteins, one abundant faster migrating species and one minor slower migrating species were detected in these cell lines and E2-dependency varied between cell lines (Figure 6A). The basal expression of the faster migrating band was significantly higher in OHTR cells compared to parental cells and was not influenced by E2 in this cell line. Note that OHTR cells do not express slower migrating FGFR2 indicating isoform switching in resistant cells. In contrast, the minor slower migrating FGFR2 was E2-inducible in the parental cell line but constitutively elevated in Ful-R cells. Therefore, anti-estrogen resistance is associated with alternative splicing of FGFR2 and corresponding change in protein levels. We used siRNA against ERα to determine which among the FGFR2 isoform expression requires ERα. ERα siRNA completely abolished the E2-inducible expression of slower migrating minor FGFR2 in parental cells and even in Ful-R cells, which has lower levels of ERα compared to other cell lines (Figure 6B). Therefore, elevated basal expression of slower migrating FGFR2 in Ful-R cells is dependent on residual ERα levels. Basal expression of faster migrating FGFR2 in OHTR cells was also ERα dependent. For unknown reason, we were only able to knockdown <50% of ERα in this single cell-derived MCF-7 clone and OHTR cells. Despite discrepancy with respect to ERα dependency of faster migrating FGFR2 in parental MCF-7 cells and clonal MCF-7 (Figure 5 and Figure 6), results support the conclusion that slower migrating FGFR2 is E2 and/or ERα dependent across cell lines.

To confirm that overexpression of FGFR2 protein in OHTR cells and lower expression in Ful-R cells correlate with mRNA changes, we performed qRT-PCR analysis (Figure 6C). OHTR cells expressed higher levels of all three FGFR2 isoforms tested, whereas Ful-R cells expressed lower levels of all three isoforms tested compared to parental cells. Therefore, FGFR2 protein changes in anti-estrogen resistant cells correlate changes in mRNAs but it is difficult to assign these changes to specific isoforms.

KGF serves as a ligand to FGFR2 C1 and C2 isoforms, whereas C3 is active independent of ligand [41,42]. Since OHTR cells expressed higher levels of FGFR2 protein compared to parental cells and Flu-R cells expressed different levels of FGFR2 isoforms compared to parental or OHTR cells, it is possible that anti-estrogen resistant cells respond differently to KGF compared to parental cells. While KGF stimulated the growth of parental cells, OHTR cells displayed elevated basal proliferation, which was unaffected upon KGF stimulation. Ful-R cells also displayed elevated basal proliferation and were unresponsive to KGF stimulation (Figure 6D). These results indicate that resistance to anti-estrogens is associated with differential FGFR2 isoform expression and response to KGF.

### FGFR2 C2 and FGFR2 C3 alter basal proliferation rate and consequently response to E2

Since FGFR2 C2 isoform expression was not E2 responsive, whereas C3 expression was repressed by E2, we examined how constitutive overexpression of these isoforms alters cellular response to E2 and tamoxifen. Expression levels of overexpressed FGFR2 isoforms are shown in Figure 6E. Both FGFR2 C2 and C3 overexpressing cells showed elevated basal proliferation compared to parental cells with retrovirus vector alone and E2 further stimulated proliferation (Figure 6F). Similar to parental cells, tamoxifen reduced E2-stimulated proliferation of FGFR2 C2 and C3 overexpressing cells. These results suggest that FGFR2 C2 and C3 overexpression provides proliferative advantage mimicking increased proliferation observed with anti-estrogen resistant cells compared to parental cells. Overall, proliferation rate of C2 or C3 overexpressing cells was much higher than parental cells (p<0.0002). Proliferation rate of FGFR2 C2 overexpressing cells remained higher than parental cells when cells were treated with E2 plus tamoxifen. Thus, individual isoforms of FGFR2 can influence basal proliferation and consequently response to E2 and anti-estrogens. Since it is difficult to knockdown individual isoforms of FGFR2 due to limited sequence divergence between isoforms and to develop isoform specific inhibitors, our observation linking specific isoforms of FGFR2 to anti-estrogen response remains correlative.

### Estrogen-induced alternative splicing of FAS and FGFR2 correlates with resistance to FAS activation-induced cell death and KGF-induced proliferation, respectively

**Figure 7:**
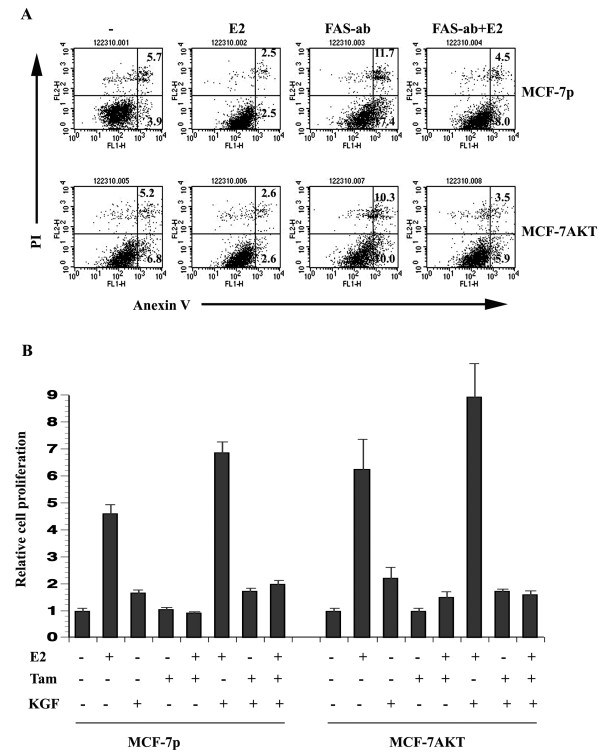
**Association between E2-induced alternative splicing of FAS and FGFR2, and cellular response to apoptosis and proliferation, respectively. A**) The effect of E2 on FAS-activation induced apoptosis. Cells were treated with 50 ng/ml FAS-activating antibody with or without co-treatment with E2 for 48 hours. Annexin V labeling followed by flow cytometry was used to measure apoptosis. Representative flow cytometry data from three experiments are shown. **B**) KGF partially overcomes the effect of tamoxifen on E2-induced cell proliferation. Cells in 96-well plate were treated with E2 (0.1 nM), 4-hydroxy tamoxifen (0.25 nM), KGF (20 ng/ml) or indicated combinations for 48 hours. BrDU-ELISA was used to measure the rate of cell proliferation. Results are from a representative of three experiments with six wells per treatment in each experiment. Reproducibly statistically significant results are described in the text.

To determine the consequences of E2-induced alternative splicing of FAS on sensitivity to FAS activation-induced cell death, we treated MCF-7p and MCF-7AKT cells with an antibody that triggers FAS-dependent apoptosis. MCF-7p and MCF-7AKT cells maintained in charcoal-stripped serum containing media displayed basal apoptosis, which was reduced upon E2-treatment (Figure 7A). FAS antibody treatment increased apoptosis in both cell types, which was more pronounced in MCF-7p compared to MCF-7AKT cells. Lower sensitivity of MCF-7AKT cells compared to MCF-7p cells is consistent with diminished basal full-length FAS protein levels in these cells. This observation is also consistent with the known anti-apoptotic function of AKT [45]. E2 substantially reduced the FAS-antibody triggered apoptosis in both cell types. Thus, E2-induced alternative splicing of FAS may have an impact on FAS-activation mediated cell death.

We next examined the effect of KGF on growth of MCF-7p and MCF-7AKT cells in the presence of E2, low dose 4-hydroxy-tamoxifen, or both. (Figure 7B). At this dose, tamoxifen reduced only E2-induced growth of both cell types. KGF increased the proliferation of both cell types (untreated versus KGF treated p=0.006 for MCF-7p and 0.0127 for MCF-7AKT). KGF plus E2 treated MCF-7p cells but not MCF-7AKT cells showed significant increase in proliferation (MCF-7p cells E2 versus E2 plus KGF, p=0.015). The proliferation rate of MCF-7p cells treated with a combination of KGF, E2, and tamoxifen were higher than untreated, tamoxifen-treated, and tamoxifen plus E2 treated cells (p<0.0002), indicating the ability of activated FGFR2 to overcome the effects of tamoxifen on E2-induced proliferation. Consistent with our previous results [6], MCF-7AKT cells treated with E2 plus tamoxifen were more proliferative than MCF-7p cells treated with E2 plus tamoxifen (p=0.021) and KGF did not additionally increase the proliferation of E2 plus tamoxifen treated MCF-7AKT cells. These results indicate E2-mediated increase in FGFR2 C1 isoform provides additional receptors for KGF allowing higher proliferation rate as well as diminished response to tamoxifen.

## Discussion

### Role and regulation of ERα in E2-regulated alternative splicing

Extracellular signal-dependent alternative splicing is a major mechanism responsible for diversification of molecular repertoire by affecting several cellular functions including apoptosis, stem cell maintenance and differentiation, cell migration, and invasion [9,46,47]. Apart from growth factor-induced signaling pathways, which can modulate alternative splicing through phosphorylation of splicing factors, steroid hormones couple transcription to splicing by recruiting splicing factors to the transcription machinery through nuclear receptors [21]. It is not known whether steroid hormones increase splicing efficiency without altering the rate of transcription. Genome wide analysis of ERα binding patterns has revealed that the largest fraction (38%) of ERα binding sites is mapped to intragenic regions including introns and several of these interactions have previously been described as unproductive [48-50]. It is possible that the primary function of ERα bound to intragenic regions is to recruit splicing factors to modulate alternative splicing without changing overall transcription rates. ERα and associated co-factors recruited to these sites may induce specific histone modifications that favor exon skipping or exon inclusion [51].

It is interesting that several of the genes that underwent E2-regulated alternative splicing have intragenic ERα binding sites (Figure 2). In fact, ~60% of the genes that bound to ERα and underwent alternative splicing without change in the rate of transcription (Additional file 1 and Additional file 4) may utilize ERα for local recruitment of histone modifying enzymes that favor alternative splicing events. We also observed both E2-induced and repressed genes undergoing alternative splicing. Thus, E2-induced alternative splicing is unrelated to rate of transcription but could be related to the ability of E2:ERα to recruit histone-modifying enzymes along the path of transcript elongation. In this context, rate of transcription elongation has greater influence on alternative splicing [51]. Alternatively, E2 inducible splicing factors such as SRSF7 is responsible for E2-induced alternative splicing without an effect of E2 on rate of transcription. SRSF7 has previously been shown to increase alternative splicing of exon 6 of BRCA1 variant with exon skipping mutation c.231G>T [52]. Therefore, genetic variation, in addition to SRSF7 induction, may contribute to diversity in E2-regulated alternative splicing events.

A large number of splicing factors associate with ERα including splicing factors of the hnRNPA1 complex (CoAA for example) and SR proteins (CAPER and SF2) [22,23,53]. Select RNA-recognition motifs (RRMs) present in these splicing factors are involved in interaction with ERα, transactivation, and splicing [53,54]. RS domains, which are present in SR proteins including SRSF7, are heavily phosphorylated by AKT [11]. Our results suggest that AKT has dominant but gene-specific effect on E2-induced alternative splicing. Activating mutation of AKT is mostly observed in ERα-positive breast cancer [26]. Therefore, it is possible that the dominant gene-specific effect of AKT on ERα:E2 induced alternative splicing is one of many functions of AKT that contribute to tumorigenesis. However, mechanistic aspect of ERα:E2 induced alternative splicing, which is additionally controlled by AKT, remain to be investigated.

### E2-regulated alternative splicing and cell signaling

To date, the specific contribution of ERα:E2-induced, -repressed, and -alternatively spliced genes on breast tumorigenesis has not been established. However, our analyses show that ERα:E2 contributes to breast cancer-enriched alternative splicing. Cancer cells often use alternative splicing as a mechanism to produce proteins that promote growth and survival [13]. Genes that we have characterized in this study for E2-regulated alternative splicing provide glimpse of how these alternative-spliced products may alter signal transduction as well as response to therapy (Figures 6 and 7).

FGFR2 alternative splicing is highly relevant as polymorphism in intron 2, which may lead to increased expression, is associated with higher risk of breast cancer [32,33]. In this context, we observed overexpression of FGFR2 in tamoxifen-resistant cells. FGFR2 C1 and C2 isoforms are ligand dependent receptors that bind to KGF, whereas the C3 isoform is constitutively active [42]. C1 isoform is expressed in normal mammary epithelial cells, whereas C3 expression is restricted to transformed cells [42]. Although all three isoforms have the transforming ability, C3 isoform is the most potent and imparts invasive capabilities [41,55]. Our results showed that E2 increases the expression of C1 isoform but reduces C3 expression in parental cells. AKT increased C1 in ERα-dependent but E2-independent manner as siRNA against ERα reduced FGFR2 levels at both basal and E2 treated condition (Figure 5). FGFR2 overexpression and isoform switching are involved in anti-estrogen resistance as FGFR2 protein species in tamoxifen and fulvestrant resistant cells are different from each other as well as from parental cells. At present, there are no assays or reagents to verify whether FGFR2 proteins differentially expressed in anti-estrogen resistant cells correspond to C1, C2, or C3 isoforms characterized in this study. Presence of at least 11 different FGFR2 isoforms, most affecting open reading frames, complicates interpretation. Nonetheless, data presented in this study suggest FGFR2 is a target for overcoming anti-estrogen resistance in breast cancer. FGFRs (both 1 and 2) are upregulated in breast cancer and cooperate with Wnt signaling to enhance mammary tumorigenesis [56]. Whether E2-induced splicing of FGFR2 and AXIN-1 (a negative regulator of Wnt) contributes to this cooperative tumorigenesis and how aberrant splicing events contribute to response to therapy remains to be investigated.

Alternative splicing of FAS is linked to apoptosis resistance in cancer [40]. FAS itself regulates alternative splicing through dephosphorylation of SR proteins [57]. FAS-activated signaling is essential for bone protective function of E2 as it is responsible for E2-induced apoptosis of osteoclasts [58,59]. Paradoxically, FAS is essential in tumor growth, although whether this function of FAS is dependent on a particular isoform is unknown [60]. The effect of E2 in preventing FAS-activation dependent apoptosis in breast cancer cells will have an impact on therapeutic response as active Fas/FasL can amplify the pro-apoptotic effects of chemotherapeutic drugs.

## Conclusions

This study identified E2 regulated alternative splicing of genes involved in apoptosis and breast epithelial cell transformation. E2-regulated splicing may be coupled to ERα:E2 regulated transcription or independent of transcription but involves ERα:E2 mediated recruitment of splicing factors/histone-modifying enzymes and/or E2 regulated expression of splicing factors such as SRSF7. Extracellular signal activated kinases such as AKT, which are often activated/overexpressed in therapy resistant breast cancers, may additionally alter these functions of E2 to modify the course of the disease. Furthermore, FGFR2 overexpression and/or alternative splicing may be associated with anti-estrogen resistance.

## Methods

### Plasmid constructs, reagents, and antibodies

CD44 minigene constructs have been described previously [21]. The constitutively active AKT (CA-AKT) and the dominant negative AKT (KD-AKT) have been described previously [6] and retrovirus constructs with CA-AKT and KD-AKT sequences were generated using the bicistronicpQCXIP vector (Clontech) [28]. FGFR2 C2 and C3 cDNAs in pBabe retrovirus vector were a generous gift from Dr. Channing Der and have been described previously [41]. ER ab10 (Neomarker, Fremont, CA) and ERα sc-543 (Santa Cruz Biotechnology, Santa Cruz, CA) were used in combination for ChIP assay. The antibody against FGFR2 was from Santa Cruz Biotechnology. Antibody against FAS (clone CH11) was from Millipore (Temecula, CA). Keratinocyte Growth Factor (KGF) was purchased from R&D Systems (Minneapolis, MN).

### Cell culture, retrovirus generation, and transfection

MCF-7 cells were grown in MEM with 10% fetal calf serum (FCS), whereas Amphophenix cells were maintained in DMEM plus 10% FCS. Retrovirus packaging and generation of MCF-7 cells overexpressing CA-AKT, KD-AKT, FGFR2 C2, and FGFR2 C3 have been described previously [28]. In all studies involving E2 treatment, cells were maintained in phenol red-free MEM plus 5% dextran charcoal treated serum (CCS) for at least four days prior to experiments and all assays were done in this media. Single cell-derived MCF-7 clone and its tamoxifen and fulvestrant resistant variants of MCF-7 cells have been described previously and were gift from Dr. Ken Nephew [44].

### ExonHit splice array

A microarray analysis of alternative splicing events covering a possible 10659 splice events of 893 apoptosis related genes was done using the array services of ExonHit Therapeutics, Inc. (Gaithersburg, MD). The splicing events analyzed include alternative splice acceptor (ASA), alternative splice donor (ASD), exon skipped (ES), exons skipped (EsS), intron retained (IR), novel exon (NE), novel exons (NEs), and partial internal exon deletion (PIED). RNA from untreated or E2 treated (10^-10^ M for 3 hours) MCF-7p and MCF-7AKT cells were assayed in quadruplicate and those probes which gave difference between untreated and E2-treated cells with a *p* value from the ANOVA analysis of less than or equal to 0.001 were considered for further analysis.

### RNA isolation and quantitative reverse transcription polymerase chain reaction (qRT-PCR)

RNA was isolated using the RNAeasy kit from Qiagen (Valencia, CA). qRT-PCR with specific primers was performed to measure the expression levels of wild type and splice variants. All qRT-PCR reactions were done using 2–5 independent RNA preparations. Mean and standard error of the mean (SE) are presented in the figures. Statistical evaluations were done using unpaired *t* test (Graphpad.com). In some cases, results from experiments that showed extreme variability were excluded. Sequences of primers used for qRT-PCR are listed in Additional file 5.

### Chromatin immunoprecipitation assay (ChIP)

ChIP-on-chip assay of MCF-7p and MCF-7AKT cells for ERα binding site has been described previously [28]. ERα binding to select genes that were alternatively spliced in response to E2 was verified by ChIP followed by real time PCR (ChIP-qPCR) as described previously [48]. The results presented are from two or more experiments. Since ChIP-qPCR is a very sensitive assay with wide experimental variability with respect to levels of E2-induced ERα binding, results of experiments that showed lower variability were selected for presentation.

### SiRNA transfection and western blotting

siRNA against ERα and the control luciferase were purchased from Dharmacon (Lafayette, CO) and transfected into cells using lipofectamine reagent. Protein extracts from transfected cells were prepared four days after transfection. Whole cell lysates from cells with or without E2 treatment for 24 hours were prepared in RIPA buffer and western blotting was preformed as described previously [61].

### Cell proliferation and cell death assays

For cell proliferation, 2000 cells maintained in phenol red-free plus 5% CCS containing media for four days were plated in the same media in each well of a 96-well plate overnight and then treated with indicated reagents for 2–6 days depending on the experiment. Bromodeoxyuridine-ELISA (EMD Chemicals, Gibbstown, NJ) was used to measure cell proliferation. For cell death assay, cells were plated in a 60 mm plate and treated with indicated reagents for 48 hours. Cell death assay was performed using Annexin V labeling followed by flow cytometry as suggested by the manufacturer (Invitrogen).

## Abbreviations

AXIN-1: Axis inhibition protein 1; CCS: Charcoal stripped serum; ChIP: Chromatin immunoprecipitation; ER: Estrogen receptor; ERE: Estrogen response element; FGFR2: Fibroblast growth factor receptor 2; hnRNP: Heterogeneous nuclear ribonuclear protein; KGF: Keratinocyte growth factor; PTB: Polypyrimidine tract binding protein; RRM: RNA-recognition motif; RT-PCR: Reverse transcription polymerase chain reaction; SMRT: Silencing mediator of retinoic acid receptor and thyroid hormone receptor; SF2: Splicing factor 2; SR proteins: Serine/arginine-rich proteins; SRSF7: Serine/arginine-rich splicing factor 7.

## Competing interests

Authors declare that they have no competing interests.

## Authors’ contribution

PN performed several of the experiments described in Figures 1, 3, 4, 5, 6 and 7. E-K.S. performed experiments described in Figure 5 and RT-PCR analysis of CD44 minigene assay. NRC characterized AXIN-1 isoforms. VNU and AKD conducted intrinsic disorder prediction of splice isoforms. BWO provided reagents used in Figure 1 and provided intellectual input for these experiments. TRG, JSC, and MB were involved in ChIP-on-chip assay of MCF-7p and MCF-7AKT cells and the analysis of data derived from this assay. HN designed most of the experiments presented in this manuscript and wrote the manuscript. All authors read and approved the final manuscript.

## Pre-publication history

The pre-publication history for this paper can be accessed here:

http://www.biomedcentral.com/1755-8794/6/21/prepub

## Supplementary Material

Additional file 1 **E2-induced splice events common to MCF-7p and MCF-7AKT cells. **This file describes E2-regulated splicing events common to MCF-7p and MCF-7AKT cells. In addition, this file also indicates ERα binding sites in the genes that undergo E2-regulated alternative splicing as well as the effect of E2 on expression of these genes in MCF-7p and MCF-7AKT cells. Furthermore, the early (three hours) and the late effects (24 hours) of E2 treatment on overall transcription of genes (extracted from NURSA website) are also shown. Longer version (LF) of the transcript in the array [reference (R) or the variant (V)] is also indicated. Positive number in fold change column indicates E2-induced splicing generating higher levels of the variant whereas negative number indicates repression of splicing event leading to generation of the variant. ASA, alternative splice acceptor; ASD; Alternative splice donor; ES, exon skipping; IR, intron retention; PEID, partial internal exon deletion. Click here for file

Additional file 2 **E2-induced splicing events observed in MCF-7p cells. **This file is similar to Additional file 1 except that splicing events unique to MCF-7p cells are indicated. Click here for file

Additional file 3 **E2-induced splicing events observed in MCF-7AKT cells. **This file is similar to Additional file 1 except that splicing events unique to MCF-7AKT cells are shown. Click here for file

Additional file 4 **Summary of ERα binding and E2-regulated expression of genes described in Additional file **1**.** Click here for file

Additional file 5 Primer sequences used for qRT-PCR analyses. Click here for file
