# Supplementary material for: Interplay between estrogen receptor and AKT in Estradiol-induced alternative splicing
Source: BMC Med Genomics. 2013 Jun 11;6:21. doi: 10.1186/1755-8794-6-21 (PMC3687557; doi:10.1186/1755-8794-6-21)
Supplement: Additional file 4 — Summary of ERα binding and E2-regulated expression of genes described in Additional file 1. [file 1755-8794-6-21-S4.doc]

| Alternatively spliced genes with ERα binding sites | | | | | | | |
| --- | --- | --- | --- | --- | --- | --- | --- |
| Total genes | Induced by E2 | | Repressed by E2 | | No effect | | Data not available |
|  | Early | Late | Early | Late | Early | Late |  |
| 89 | 18 | 15 | 16 | 22 | 50 | 47 | 5 |
| Alternatively spliced genes without ERα binding sites | | | | | | | |
| 64 | 13 | 19 | 4 | 10 | 43 | 31 | 4 |
