# Supplementary material for: Interplay between estrogen receptor and AKT in Estradiol-induced alternative splicing
Source: BMC Med Genomics. 2013 Jun 11;6:21. doi: 10.1186/1755-8794-6-21 (PMC3687557; doi:10.1186/1755-8794-6-21)
Supplement: Additional file 5 — Primer sequences used for qRT-PCR analyses. [file 1755-8794-6-21-S5.doc]

| Gene name | Forward primer sequence | Reverse primer sequence |
| --- | --- | --- |
| qRT-PCR primers | | |
| AXIN-1a | 5’ TGC AGG AGG TTA TGC GGC GG 3’ | 5’ GCT CCA TGT CCG ACA CGG CT 3’ |
| AXIN-1b | 5’ CAG AGG ACA AGA TCG CAG AG 3’ | 5’ GGG TTC CCC GCA GAA CTA GT 3’ |
| Total Fas | 5’ GGT ACT GAA CAG GCA GGC CAC TTT 3’ | 5’ TGC AAA GGT CTT TGA GGT AGA GCC 3’ |
| Isoform Δ 3,4 | 5’ CCC TGT CCT CCA AGA TGT GAA CAT G 3’ | 5’ CCC AAA CAA TTA GTG GAA TTG 3’ |
| FGFR2 C1 | 5’AAG AAG GTT TCT GGA GCA GTG GAC 3’ | 5’ TTA ACA AGG AAG GCA GAA CGC ACG 3’ |
| FGFR2 C2 | 5’ACT CTT CTC TTC CCT TCT TTC AGG 3’ | 5’ TGG GTC AGG ATA ACA AGG TGA ATA CG 3’ |
| FGFR2 C3 | 5’ TTG GTA GAA GAC TTG GAT CGA AT 3’ | 5’ GGC TGG AAT GCA ATG GCA TGA TCT 3’ |
| CD44 Exon-I | 5’ CAA GGT GAA CGT GGA TGA AG 3’ | 5’ AGC ACT TCC GGA TTT GAA TG 3’ |
| CD44 Exon-S | 5’ GGC CCT GGG CAG GCT GTT TGG 3’ | 5’ GAG CCT TCA CCT TAG GGT TG 3’ |
| SRSF7 | 5’ CAG ATA CCT AGG CTT GTC CAT TT 3’ | 5’ CTA GCG GTC AAA CTA CCG AAT AA 3’ |
| βActin | 5’ AAT GRG GCC GAG GAC TTT GAT TGC 3’ | 5’ AGG ATG GCA AGG GAC TTC CTG TAA 3’ |
| ChIP-PCR primers | | |
| AXIN-1 | 5’ AGC CTA GCA ACC AAA GTG TC 3’ | 5’ TGG GTT TAA CTG TGA CCT CCT GC 3’ |
| Caspase 7 | 5’ TGC GTG TGT GTG TGT GTT 3’ | 5’ ACA CAG GCA TTC CAG TTC AC 3’ |
| FAS | 5’ TGA GCA GTT CGG TTT GGA GCA AAG 3’ | 5’ TTG GCA CAA GAC GCA CTC TAG TCA 3’ |
| FGFR2 | 5’ AGC TGG GAT TAC AGG CAT GTA CCA 3’ | 5’ TGA CCA GCG TGA TGA AAC CAT GTG 3’ |
